# Tuberculosis household contact tracing in children: axes of inequality, Barcelona 2003–2022

**DOI:** 10.3389/fpubh.2025.1591552

**Published:** 2025-05-29

**Authors:** Raquel Prieto-García, Joan Pau Millet, Antonio Soriano-Arandes, María Espiau, Claudia Broto, Mar Ronda, Núria López, Antoni Noguera-Julian, Eva Masdeu Corcoll, Cristina Domingo Jimenez, Miriam Ros Samsó, Maria Isabel Marcos Arroita, Jesús Edison Ospina Valencia, Carmen García Rebollo, Pere Simon Viván, Cristina Rius Gibert

**Affiliations:** ^1^Departament de Ciències Experimentals i de la Salut (DCEXS). Universitat Pompeu Fabra, Barcelona, Spain; ^2^Servei d’Epidemiologia (SEPID), Agència de Salut Pública de Barcelona (ASPB), Barcelona, Spain; ^3^Institut de Recerca de l'Hospital de la Santa Creu i Sant Pau (IIB Sant Pau), Barcelona, Spain; ^4^CIBER de Epidemiología y Salud Pública (CIBERESP), Barcelona, Spain; ^5^Unitat de Patologia Infecciosa i Immunodeficiències de Pediatria, Hospital Universitari Vall d'Hebron, Barcelona, Spain; ^6^Departament de Malalties Infeccioses, Hospital Universitari de Bellvitge, l’Hospitalet del Llobregat, Barcelona, Spain; ^7^Servicio de Pediatría, Hospital del Mar, Barcelona, Spain; ^8^Malalties Infeccioses i Resposta Inflamatòria Sistèmica en Pediatria, Servei de Malalties Infeccioses i Patologia Importada, Institut de Recerca Pediàtrica Sant Joan de Déu, Barcelona, Spain; ^9^Centro de Investigación Biomédica en Red de Epidemiología y Salud Pública (CIBERESP), Madrid, Spain; ^10^Departament de Cirurgia i Especialitats Medicoquirúrgiques, Facultat de Medicina i Ciències de la Salut, Universitat de Barcelona, Barcelona, Spain

**Keywords:** latent tuberculosis infection, contact tracing, children, axes of inequality, tuberculosis

## Abstract

Children under 15 years of age living in the household of a tuberculosis case constitute a very vulnerable group to tuberculosis infection (TBI). The objective of this study was to determine the prevalence of TBI and the risk factors associated with presenting TBI in this group, considering sex, age, and migratory status as axes of inequality. A population-based, analytical, cross-sectional observational study was carried out in the city of Barcelona in the period 2003–2022. The study population was household contacts under 15 years of age with index cases of pulmonary TB reported to the Barcelona Public Health Agency in the period 2003–2022. The analyses were performed using Generalized Estimating Equations (GEE) to predict the risk of TBI among these cohabiting contacts and were stratified considering the inequality axes of sex and migratory status. A total of 1084 contacts under 15 years of age were studied from 693 cases of tuberculosis. TBI prevalence among contacts was 24.5%. The factors associated with the presence of TBI in the contacts were having a smear positive in the index case, being older than 5 years in the contacts ([5,10], [10–15]) and the case and the contact being migrants; smear positive when the index case was native women and being from a municipal district with a lower incidence of tuberculosis when the index case was native women and the men. The results of the study confirm the importance of carrying out contact tracing and follow-up of household children, especially if the index case is smear positive. Contact tracing should be carried out as soon as possible to assess the prescription of primary chemoprophylaxis and TBI treatment to avoid rapid TB progression in children.

## Introduction

Tuberculosis (TB) was declared a public health emergency in 1993 by the World Health Organization (WHO) and still poses a significant global health challenge (1). In 2021 in Spain, the incidence of TB was 7.4 cases per 100,000 inhabitants in the whole population, and 4.1 cases per 100,000 among children under 15 (2). In 2022 in Barcelona, the incidence of TB among children under 15 was 5.3 cases per 100,000 (3). In Barcelona, the current measures include the implementation of the Barcelona Tuberculosis Prevention and Control Program, which focuses on early diagnosis, epidemiological surveillance, contact tracing, and treatment management. However, significant challenges remain, such as drug resistance, the identification and treatment of latent infections, and the need to improve intersectoral coordination, especially in vulnerable populations such as children.

TB transmission is primarily airborne and depends on factors like cohabitation and shared activities with the index case (IC), the bacillary load and the disease severity of the IC, and poor home ventilation, among others (3–5). The progression from initial TB infection to TBI involves the immune response. *Mycobacterium tuberculosis* enters a dormant state due to factors like a weakened immune system as occurs in children. Unlike acute TB infection, individuals with TBI do not exhibit symptoms and cannot transmit the disease. This distinction is crucial. Active TB is characterized by symptoms like persistent cough, chest pain, and weight loss, and can spread to others. The burden of TBI is less than one-quarter of the world’s population (6). It is estimated that 5–10% of individuals develop TB within 2 to 5 years from infection (7–9), with the risk being even higher in children under the age of five (3, 10). To reduce this risk, contact tracing and treatment of latent tuberculosis infection (TBI) are essential measures recommended by the WHO’s End-TB Strategy for TB elimination (11–13). These should begin immediately after diagnosis of the IC, especially if the IC has a high bacillary load (14, 15).

Barcelona, like other large European cities, has extensive social heterogeneity, with vulnerable populations concentrated in specific neighborhoods where health inequalities are significant (16). These inequalities stem directly from urban health determinants, such as governance, physical context, socioeconomic factors, and physical environment. In addition, these determinants can affect people differently depending on several axes of inequality including sex, age, and migratory status (17). These axes are critical for understanding the disparities in the burden of TBI and for designing targeted interventions to improve TB prevention and control in the most vulnerable populations.

This study aimed to determine the prevalence and risk factors of TBI in children under 15 years old living with an IC in Barcelona from 2003 to 2022, considering axes of inequality: sex, age, and migratory status.

## Methods

### Study design and population

We conducted a population-based cross-sectional analytical observational study in the city of Barcelona from 1 January 2003 to 31 December 2022. This study was a secondary analysis of routinely collected data.

The study included household contacts under 15 years of age of ICs with pulmonary TB infection reported to the ASPB in the period 2003–2022. The exclusion criteria for these contacts were having had a history of TB or TBI. Contacts were removed from the study if they did not attend the tuberculin skin test (TST) or the TST reading or were lost of follow-up in the tuberculosis unit if the test was positive.

### Contact tracing and testing procedure

The Epidemiology Service of the ASPB carried out the contact tracing in collaboration with community health agents. We conducted a structured interview with the TB case and the identified contacts as a part of routine surveillance, prevention, and control activities from the Barcelona TB Prevention and Control Program. The TST was performed on the latter, considering positive those who presented an induration larger than 5 mm. If the result was negative, a second TST was performed 8–12 weeks later. All study data were recorded and analyzed anonymously. Access to these data, for subsequent analysis, was carried out between November 3, 2023, and March 30, 2024, and at the time of data extraction, they were anonymized so that the people who carried out the statistical analysis did not have access to information that could individually identify the participants.

Contacts with a positive TST or symptoms compatible with TB were referred to one of the designated TB clinical units in Barcelona where they underwent a chest X-ray and interferon-gamma release assay based on medical criteria. These units also managed any necessary treatment and follow-up for TBI or active TB.

### Variables

The explanatory variables of the IC were sex, migratory status, age, municipal district (MD) of residence, and smear positive. The MD variable was classified into two categories based on the prevalence of TB in Barcelona: high prevalence with more than 10 cases per 100,000 inhabitants (MD of Ciutat Vella, Sant Martí, and Nou Barris districts) and low prevalence with less than or equal to 10 cases per 100,000 inhabitants (MD of Sants-Montjuïc, Sant Andreu, Eixample, Horta-Guinardó, Gràcia, Les Corts, and Sarrià districts) (18).

The explanatory variables of the contacts were sex, age, and migratory status. The stratification of age groups into 3 categories ([0–5], [5–10], [10–15]) was done to reflect key stages of child development and TB risk, consistent with prior pediatric TB literature and clinical practice. Children under 5 are considered at higher risk for rapid progression and severe forms of TB, often guiding prioritization in contact tracing and preventive treatment. The [5–10] and [10–15] age groups were defined to reflect school-age and early adolescent populations, which differ in terms of exposure patterns, immunological maturity, and likelihood of adherence to preventive treatment (19, 20). The migratory status and the MD variables serve as reliable indicators for determining socioeconomic level.

The response variable was the diagnosis of TBI, recorded as either present or absent for each investigated contact.

### Statistical analysis

We performed a descriptive analysis of the sociodemographic and clinical characteristics of the IC and the sociodemographic variables of the contacts. The age distribution of the ICs was analyzed using the Shapiro–Wilk test, as it did not follow a normal distribution. Therefore, for this variable, we used the median as a measure of central tendency and the interquartile range as a measure of dispersion. For all the analyses, the ICs were stratified by sex and migratory status. In all tests, the level of significance was set at 0.05.

We used the Pearson chi-squared test to determine the association between qualitative variables and the prevalence of TBI among contacts, and the Mann–Whitney U test to determine the association between quantitative variables and the prevalence of TBI among contacts.

Considering that our outcomes were not independent across contacts of the same IC, we used Generalized Estimating Equations (GEE) (21), an extension of generalized linear models that allows regression analysis of outcomes that may not be normally distributed or independent. We performed a bivariate analysis to select the demographic variables of ICs and contacts that best predict the risk of TBI among contacts. The variables showing statistical significance were included in the stratified multivariate analysis.

We used the statistical program RStudio Version 1.3.1093.

### Ethical considerations

Demographic and clinical data were obtained from the epidemiological questionnaire used by the Barcelona Tuberculosis Prevention and Control Program. All study data were recorded and analyzed anonymously. Data were collected routinely according to the National Tuberculosis Plan approved by the Spanish Ministry of Health and the statistical analysis was carried out retrospectively between March 1, 2023, and September 30, 2023. At the time of data extraction, people who performed the analysis did not have access to information that could individually identify the participants. Therefore, informed consent was not required. The project was approved by the Parc de Salut Mar Drug Research Ethics Committee (CEIm-PSMAR, 2023/11081). All data were treated strictly confidential under the regulations: the ethical principles of the Declaration of Helsinki (22); the Organic Law 7/2021 (23), of May 26, on the protection of personal data processed for prevention, detection, investigation and prosecution of criminal offenses and the execution of criminal penalties; and the Directive (EU) 2016/680 of the European Parliament and of the Council, April 27, 2016 (24).

## Results

**Figure 1:**
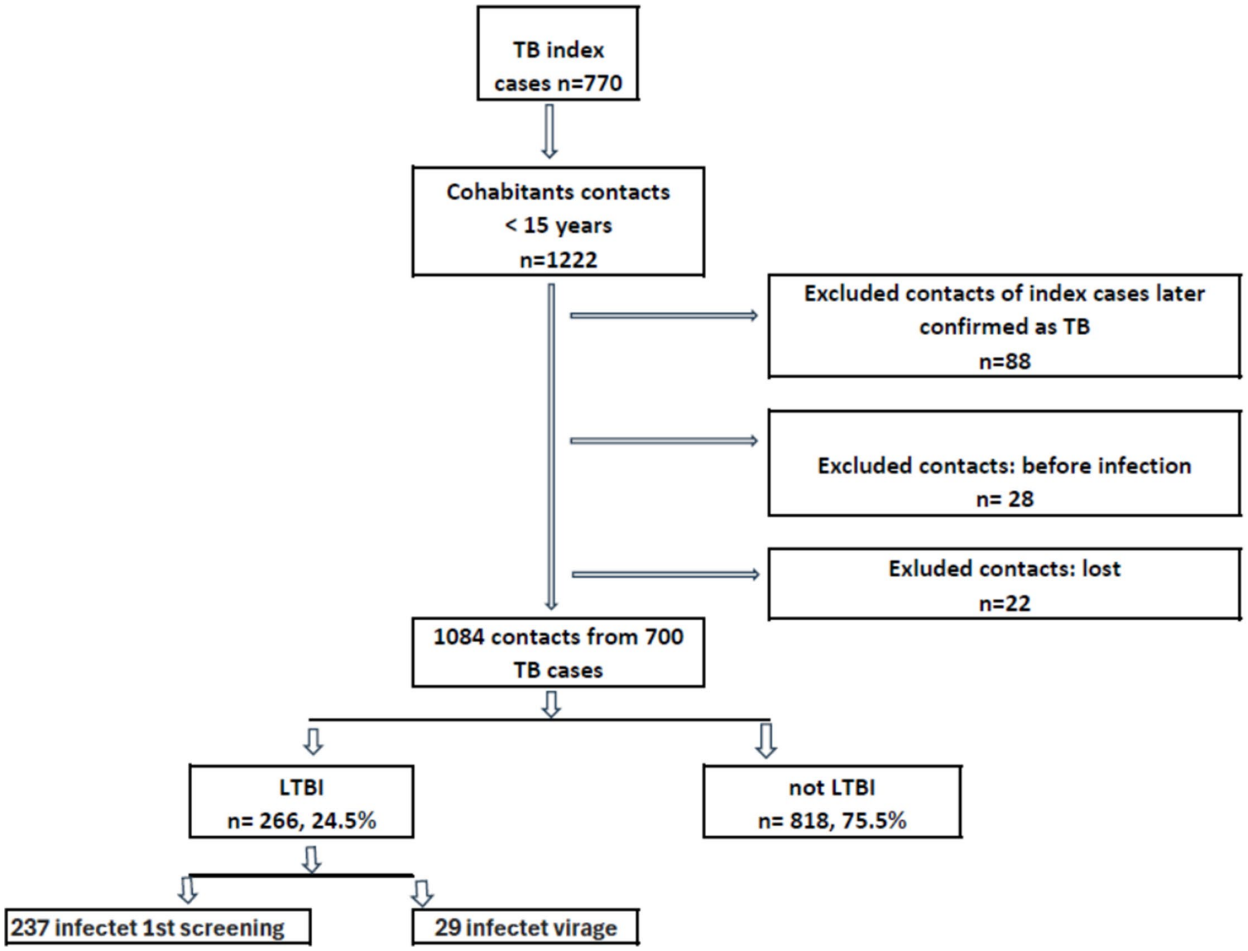
Flowchart of tuberculosis cases and their cohabiting contacts younger than 15 years reported to the Epidemiological Surveillance Service of the Public Health Agency of Barcelona. Barcelona, 2003–2022.

**Figure 2:**
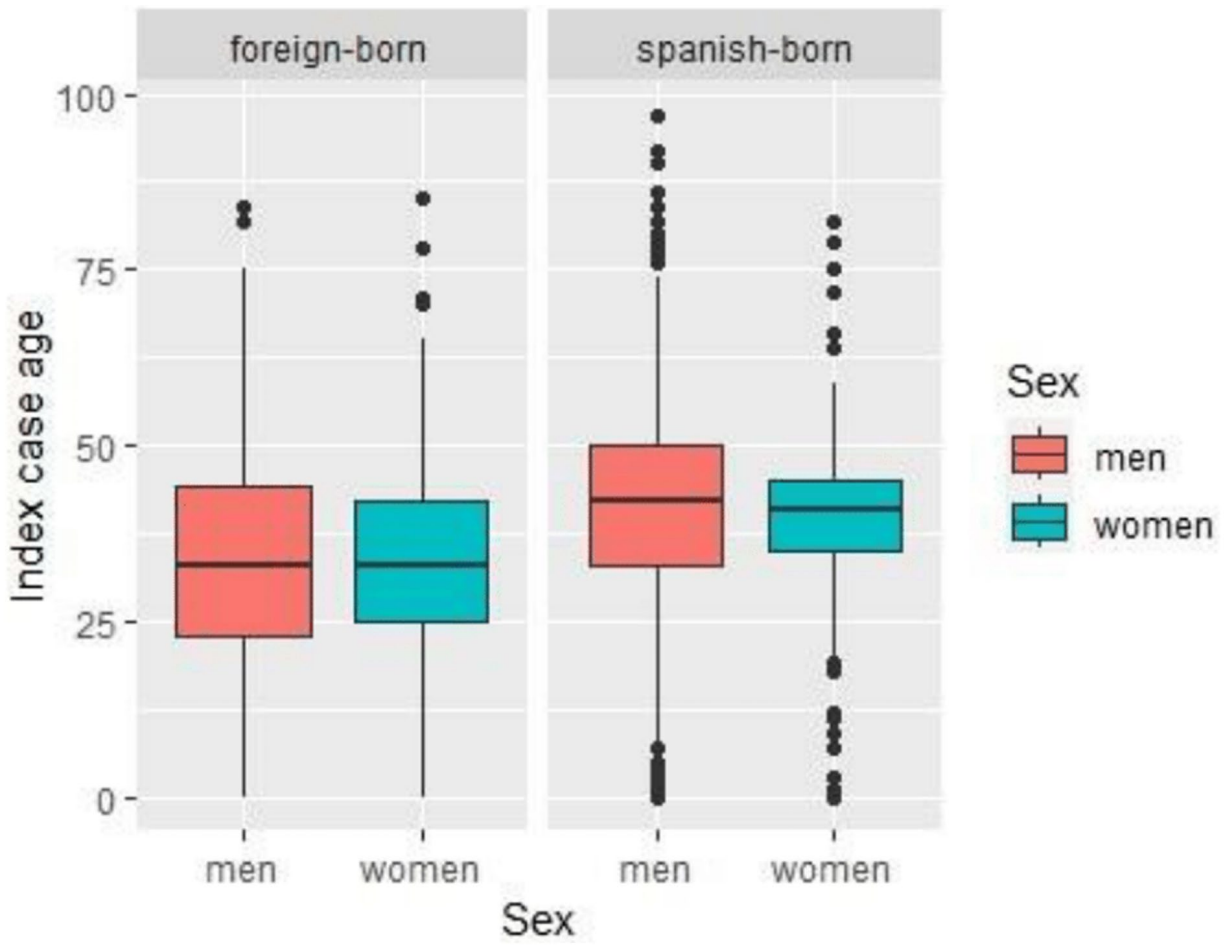
Distribution of ICs age by sex in foreign-born (left) and Spanish-born (right). Age distribution is shown in males (red) and females (teal) ICs. The median age, interquartile range, and outliers are displayed for each group.

In Barcelona, between January 2003 and December 2022, 770 cases of pulmonary TB were reported with 1,222 cohabiting contacts under 15 years of age. During the study, 22 (1.8%) of these contacts were lost to follow-up and were therefore removed from the statistical analysis. After applying the selection criteria, the study population included 1,084 contacts of 700 TB cases. The number of contacts who tested positive for the TST was 237 at the first screening and 29 at the second screening, for a total of 266 (Figure 1). The median age of TB IC with contacts with TBI was lower in migrants, both for females and males (Figure 2).

**Figure 3:**
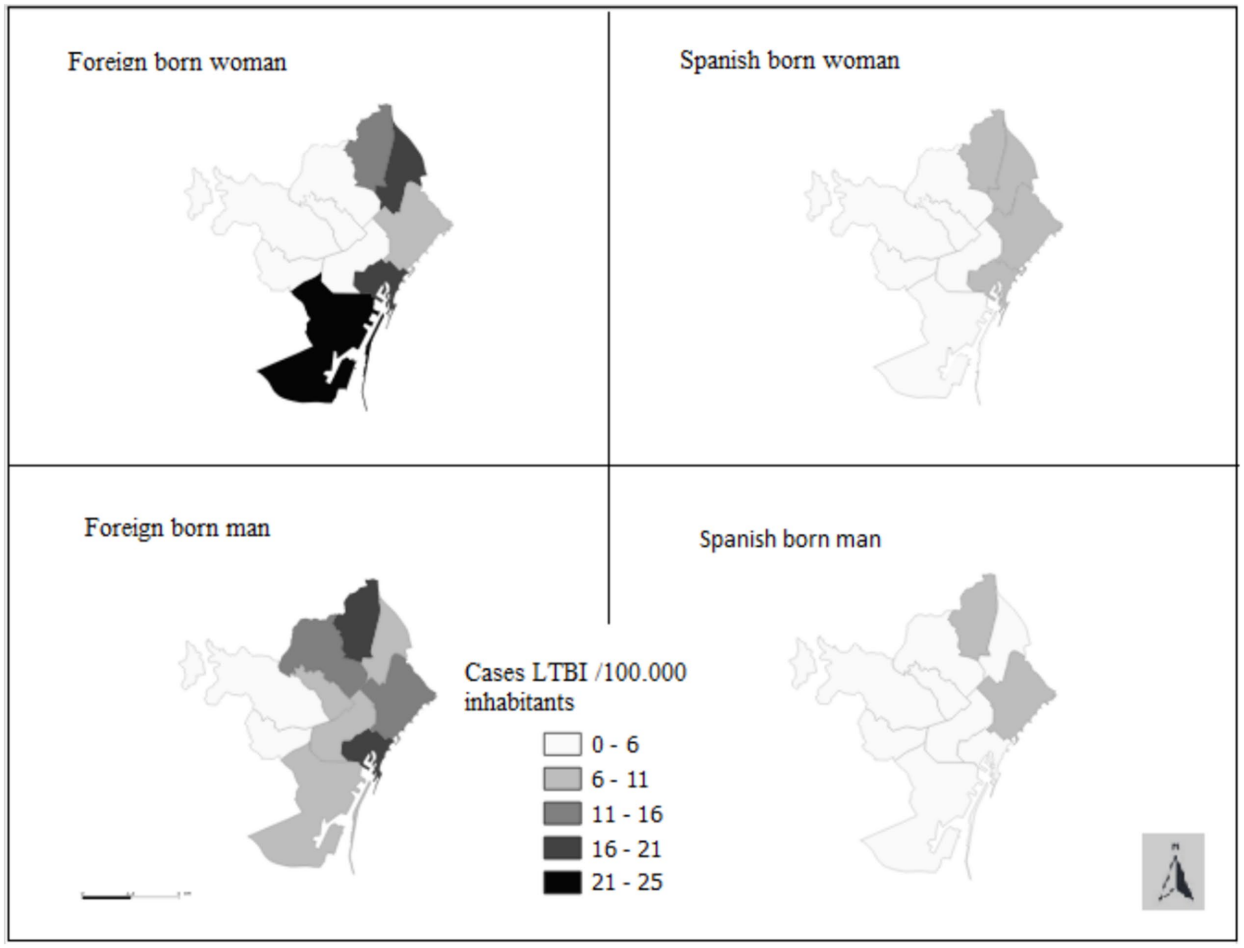
Distribution of TBI Cases Among Contacts by Residential MD of the Index Case, Stratified by Sex and Migratory Status (Barcelona, 2003–2022).

Figure 3 illustrates the geographic distribution of TBI cases according to the MD of residence of the IC without statistical significance.

The mean number of cohabiting contacts under 15 years of age per case was 1.61 when the IC was a female and 1.49 when the IC was a male (Tables 1.1, 1.2). Among contacts, the prevalence of TBI was 24.5% (Tables 1.1, 1.2) and the prevalence of TB was 7.5%.

**Table 1.1:** Characteristics and TBI prevalence among contacts of female ICs, by IC and contact demographics, Barcelona 2003–2022.

Variables	Categories	Index case female (*N* = 342)			
		Foreign-born (*N* = 210)		Spanish-born (*N* = 132)	
		TBI n (%)	*p*-value*	TBI n (%)	*p*-value*
*n* = 549 (1.61 contacts / case)		**87 (8%)**		**40 (3.7%)**	
Index case characteristics					
Age (median; IQR) 1		35 (28–43)	0.14**	40 (34–42)	0.09**
MD	Low	55 (63.2)	**0.019**	18 (45.0)	**0.006**
	High	32 (36.8)		22 (55.0)	
Smear positive	No	23 (26.4)	**<0.001**	19 (47.5)	**0.010**
	Yes	64 (73.6)		21 (52.5)	
Contact characteristics					
Sex	Woman	38 (43.7)		22 (55.0)	0.564
	Man	49 (56.3)	0.411	18 (45.0)	
Age	[0–5]	15 (17.2)	**<0.001**	0 (0.0)	**<0.001**
	[5–10]	28 (32.2)		19 (47.5)	
	[10–15]	44 (50.6)		21 (52.5)	
Contact country	Spanish-Spanish		**<0.001**	36 (90.0)	0.541
	Spanish-Foreign			4 (10.0)	
	Foreign-Spanish	27 (31.0)			
	Foreign-Foreign	60 (69.0)			

*Pearson Chi-square test; ^Fisher’s exact test; 1. Shapiro–Wilk normality test; **U Mann Whitney. Values in bold indicate statistical significance (*p* < 0.05).

**Table 1.2:** Characteristics and TBI prevalence among contacts of male ICs, by IC and contact demographics, Barcelona 2003–2022.

Variables	Categories	Index case male (*N* = 358)			
		Foreign-born (*N* = 224)		Spanish-born (*N* = 134)	
		TBI n (%)	*p*-value*	TBI n (%)	*p*-value*
*n* = 535 (1.49 contacts / case)		**100 (9.2%)**		**39 (3.6%)**	
Index case characteristics					
Age (median; IQR) 1		32 (20–41)	0.18**	42 (38–45)	0.49**
MD	Low	46 (46.0)	1.000	17 (43.6)	0.096
	High	54 (54.0)		22 (56.4)	
Smear positive	No	34 (34.0)	**<0.001**	18 (46.2)	0.715
	Yes	66 (66.0)		21 (53.8)	
Contact characteristics					
Sex	Woman	39 (39.0)		20 (51.3)	0.587
	Man	61 (61.0)	0.052	19 (48.7)	
Age	[0–5]	11 (11.0)	**<0.001**	5 (12.8)	0.215
	[5–10]	38 (38.0)		12 (30.8)	
	[10–15]	51 (51.0)		22 (56.4)	
Contact country	Spanish-Spanish		**<0.001**	37 (94.9)	1.000
	Spanish-Foreign			2 (5.1)	
	Foreign-Spanish	24 (24.2)			
	Foreign-Foreign	75 (75.8)			

*Pearson Chi-squared test; 1. Shapiro–Wilk normality test; **U Mann Whitney; 2. Pearson Chi-squared test for each category. Values in bold indicate statistical significance (*p* < 0.05).

Among the contacts of female ICs, the variables significantly associated with TBI were a smear positive of the IC and an age range of 10 to 15 years of the contacts (Table 1.1). In the case of migrant female ICs, other variables significantly associated with TBI were the following: MD with a high prevalence of TB, and the concomitant migrant status of the case and the contact. In the case of native female ICs, there was a significant association between TBI and living in an MD with a low prevalence of TB (Table 1.1; Figure 3).

Among the contacts of migrant male ICs, the variables significantly associated with TBI were a smear positive, an age range of 10 to 15 years, and the concomitant migrant status of the case and the contact (Table 1.2). In the case of native male ICs, none of the variables studied presented significant differences (Table 1.2). Among migrants, both females and males and especially when the IC was a male, we observed that the older the age of the contact, the greater the presence of TBI.

In the bivariate analysis, among the contacts of migrant ICs, both females and males, the following factors were significantly predicting TBI: a smear positive of the IC, older contact age, and the migratory status of both the IC and the contact. For contacts of native ICs, both females and males, TBI was predicted by residing in an MD with a lower incidence of TB and smear positive when the ICs were native females.

The multivariate analysis confirmed the results of the bivariate analysis, except for MD in the case of native female ICs (Tables 2.1, 2.2). As we describe in the bivariate analysis, among the contacts of migrant ICs, both females and males, the following factors were significantly predictive: smear positive of the IC and older contact age. For contacts of native females’ ICs, the migratory status of both the IC and the contact was significant too.

**Table 2.1:** Multivariate model (GEE) predicting TBI risk among contacts of female ICs, by IC and contact demographics.

Variables	Categories	Index case women			
		Foreign-born		Spanish-born	
		ORc (95% CI)	ORa (95% CI)	ORc (95% CI)	ORa (95% CI)
Index case characteristics					
Age		1.02 (1.00–1.04)	1.02 (1.00–1.04)	0.98 (0.96–1.00)	0.98 (0.96–1.00)
MD	Low	1		1	1
	High	0.57 (0.32–1.04)		**2.41 (1.06–5.46)**	2.10 (0.91–4.83)
Sputum smear positive	No	1		1	1
	Yes	**3.12 (1.68–5.80)**	**3.38 (1.74–6.54)**	**3.04 (1.32–6.97)**	**3.03 (1.29–7.12)**
Contact characteristics					
Sex	Woman	1		1	
	Man	1.30 (0.79–2.12)		0.85 (0.43–1.65)	
Age	[0–5]	1		1	
	[5–10]	**2.45 (1.34–4.47)**	**2.14 (1.16–3.98)**	0	
	[10–15]	**4.25 (2.30–7.83)**	**2.99 (1.54–5.79)**	inf	
Contact country	Spanish-Spanish			1	
	Spanish-Foreign			1.92 (0.41–9.04)	
	Foreign-Spanish	1			
	Foreign-Foreign	**3.45 (1.95–6.10)**	**2.46 (1.31–4.59)**		

Crude and adjusted Odds Ratio (OR) with 95% Confidence Intervals (95% CI) are shown. Barcelona. 2003–2022. CI, confidence interval; ORc, crude odds ratio; ORa, adjusted odds ratio. Values in bold indicate statistical significance (*p* < 0.05).

**Table 2.2:** Multivariate model (GEE) predicting TBI risk among contacts of male ICs, by IC and contact demographics.

Variables	Categories	Index case men			
		Foreign-born		Spanish-born	
		ORc (95% CI)	ORa (CI 95%)	ORc (95% CI)	ORa (95% CI)
Index case characteristics					
Age		1 (0.98–1.02)	1.01 (0.99–1.02)	1.00 (0.98–1.01)	0.99 (0.98–1.01)
MD	Low	1		1	
	High	0.81 (0.47–1.41)		**2.40 (1.05–5.47)**	**2.42 (1.06–5.56)**
Smear positive	No	1		1	
	Yes	**2.32 (1.32–4.07)**	**2.44 (1.39–4.28)**	1.31 (0.59–2.94)	
Contact characteristics					
Sex	Woman	1		1	
	Man	1.42 (0.89–2.24)		0.89 (0.51–1.57)	
Age	[0–5]	1		1	
	[5–10]	**3.69 (2.00–6.82)**	**3.73 (1.95–7.12)**	1.80 (0.88–3.70)	
	[10–15]	**4.04 (2.03–8.02)**	**4.28 (2.04–8.98)**	2.07 (0.93–4.61)	
Contact country	Spanish-Spanish			1	
	Spanish-Foreign			1.18 (0.38–3.67)	
	Foreign-Spanish	1			
	Foreign-Foreign	**2.30 (1.37–3.85)**	1.67 (0.94–2.97)		

Crude and adjusted Odds Ratio (OR) with 95% Confidence Intervals (95% CI) are shown. Barcelona 2003–2022. CI, confidence interval; ORc, crude odds ratio; ORa, adjusted odds ratio. Values in bold indicate statistical significance (*p* < 0.05).

We also performed an analysis of the age distribution of contacts that developed TB compared to the ones presenting TBI. This analysis found that younger age was directly associated with the presence of TB in the case of the migrant and native female ICs and the migrant male ICs (Tables 3.1, 3.2).

**Table 3.1:** Age distribution of contacts of female ICs, stratified by migratory status, Barcelona 2003–2022.

Variables	Categories	Index case female					
		Foreign-born			Spanish-born		
		TB n (%)	TBI n (%)	*p*-value*	TB n (%)	TBI n (%)	*p*-value*
n		30	87		12	40	
Contact characteristics							
Age	[0–5]	16 (53.3)	15 (17.2)	**<0.001**	6 (50.0)	0 (0.0)	**<0.001**
	[5–10]	10 (33.3)	28 (32.2)		4 (33.3)	19 (47.5)	
	[10–15]	4 (13.3)	44 (50.6)		2 (16.7)	21 (52.5)	

*Pearson Chi-squared test. Values in bold indicate statistical significance (*p* < 0.05).

**Table 3.2:** Age distribution of contacts of male ICs, stratified by migratory status, Barcelona 2003–2022.

Variables	Categories	Index case male (*N* = 358)					
		Foreign-born			Spanish-born		
		TB n (%)	TBI n (%)	*p*-value*	TB n (%)	TBI n (%)	*p*-value*
n		34	100		12	39	
Contact characteristics							
Age	[0–5]	19 (55.9)	11 (11.0)	**<0.001**	3 (25.0)	5 (12.8)	0.532
	[5–10]	10 (29.4)	38 (38.0)		4 (33.3)	12 (30.8)	
	[10–15]	5 (14.7)	51 (51.0)		5 (41.7)	22 (56.4)	

*Pearson Chi-squared test. Values in bold indicate statistical significance (*p* < 0.05).

**Figure 4:**
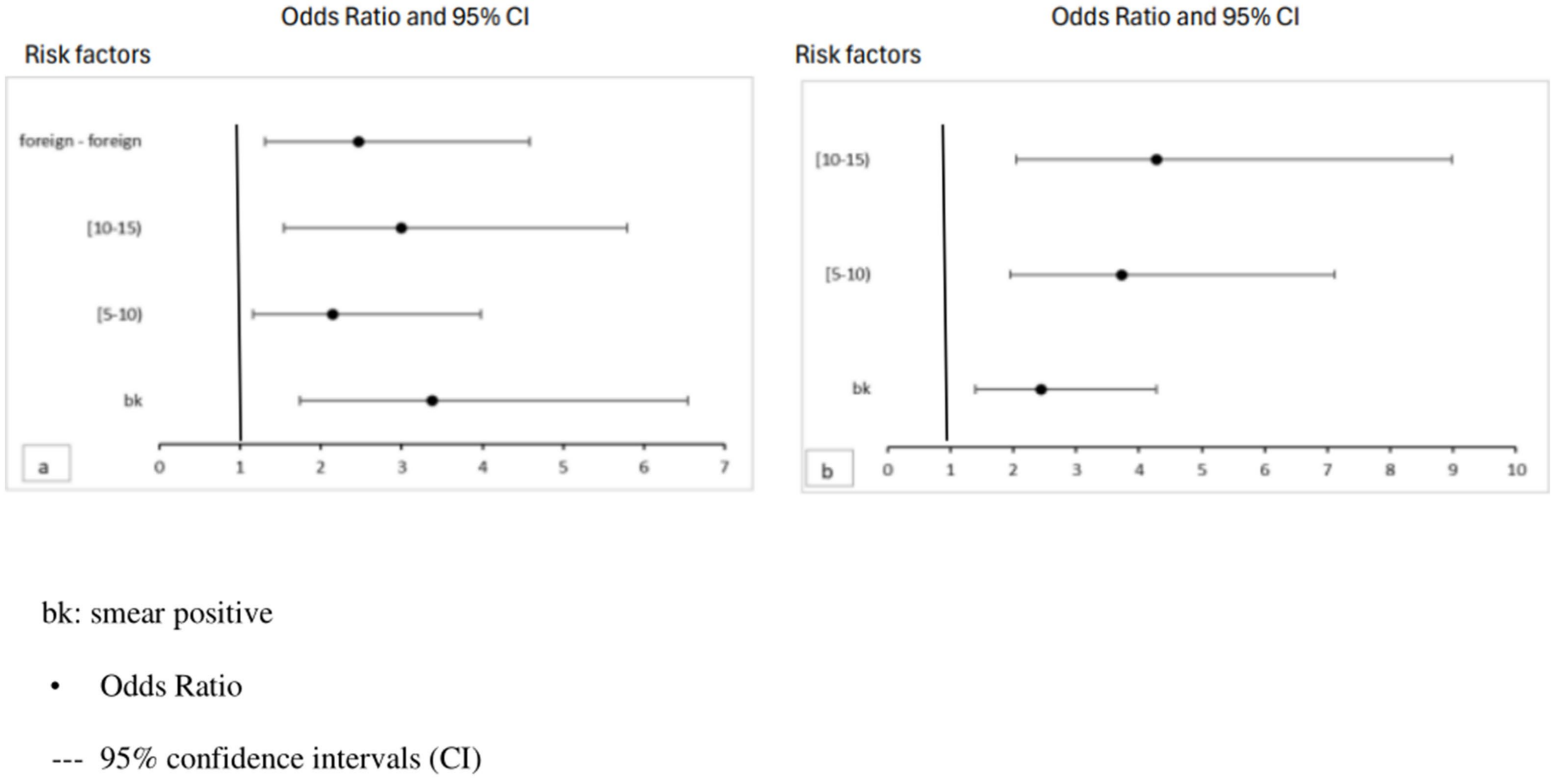
Risk factors for TBI in contacts of foreign-born female **(a)** and male **(b)** ICs by age. Data are presented as odds ratios with 95% CIs.

Figure 4 illustrates the results of the multivariate analysis among contacts of migrant ICs, females and males, according to the significant variables.

## Discussion

During the study period (2003–2022), the prevalence of TBI among cohabiting contacts of TB ICs under 15 years of age was 24.5%, higher than the estimated 3% among the population under 15 years of age in Europe (1) and similar to the 27.9% found in contacts under 5 years of age in a systematic review of 17 studies in low TB burden countries (25). Other research in high-income countries has reported TBI prevalence in children under 15 years old ranging from 16 to 41% (26). The mean age of ICs was highest in male natives, followed by female natives, migrant males, and migrant females.

Contacts of native male ICs from MDs with lower TB incidence were at higher risk of TBI compared to other contacts. This elevated risk may be related to delays in the diagnosis and treatment of these ICs, possibly because of limited awareness and perception of TB risk in ICS males (21). Additionally, males may be influenced by social constructs of strength, leading to delays in seeking medical care. They are less likely to report their symptoms and may reject interventions, particularly in the context of low-qualified and low-wage informal jobs, and of the idealization of harmful masculinities (27, 28). Similarly, in the unadjusted analysis, contacts of native female ICs from MDs with lower TB incidence showed a higher risk of TBI, although this was not statistically significant.

Contacts of smear positive TB patients, likely at a more advanced disease stage, had a higher risk of developing TBI. This risk was highest among contacts of migrant female ICs, followed by contacts of native female ICs and contacts of migrant male ICs. This diagnostic delay for female ICs could be linked to gender-related health inequalities, most likely due to their role as primary family caregivers (29, 30). This has been observed in national health surveys (31) and qualitative studies conducted in high TB burden settings (32). This delay is even higher among migrant women, for whom the interrelated challenges of being a woman and a migrant exacerbate the situation, according to other studies (33).

Another factor contributing to delayed diagnosis is the stigma associated with TB, which may lead ICs to fear job loss, eviction, or restricted access to education. Stigma, as a social determinant of health, also creates barriers to accessing health care systems (34). For migrant populations, these barriers contribute to increased diagnostic delays and increased transmission of TB within the community and among close contacts, as reported in previous studies (35). Additional research has linked TB to a lack of family support, arising from fears of infection and social blame, further exacerbating diagnostic delays (36).

Contacts of migrant men with IC, followed by contacts of migrant women with IC in contacts older than 5 years, showed a higher risk of TBI, with a positive gradation of risk. This same gradation has been observed in other studies (37) and may be explained by greater exposure to possible TB cases in the contacts’ countries of origin, although this explanation has not been verified. To explore this hypothesis, in the future, the time elapsed between the contacts’ arrival in the country and their diagnosis could be evaluated in different age groups.

Being both the IC and the contact migrants represented a risk factor for TBI in contacts, possibly because of increased exposure from travel to their countries of origin (6, 38).

One limitation of this study is the exclusion or inaccuracies in data collection of some important variables, such as diagnostic delay, symptom presence, and income. Additionally, the data obtained in this study may overestimate TBI prevalence, as in many contacts the screening of migrated patients has not been carried out as required by the 2015 “Recommendations for the prevention and control of pediatric tuberculosis in Catalonia” (6, 39).

Another limitation of our study is that some stratification resulted in small groups, and this could lead to a loss of statistical power in the multivariate analysis.

This population-based study’s findings could be extrapolated to other large cities with high migrant populations and similar TB burden and incidence profiles to Barcelona. Such population characteristics are likely to become increasingly common because of demographic changes and displacements driven by climate change (40).

Although not specifically analyzed in our study, effective contact studies in migrant populations require support from community health agents, especially in groups facing strong language barriers (41).

The results of our study underscore the importance of contact studies to promptly detect and treat TBI in children under 15 years of age, aiming to prevent progression to active TB. Our study supports the current recommendations for contact tracing in pulmonary TB ICs. It also highlights the need for additional measures, such as providing primary chemoprophylaxis for contacts under 5, patient and family education, home visits, minimizing delays in diagnosing ICs, conducting active case surveillance, and addressing axes of inequality (sex, age, and migratory status), treatment monitoring, and the involvement of the health system, especially tuberculosis units (42). Incorporating these strategies can improve the effectiveness of contact tracing and ensure proper follow-up for treatment adherence.

## Data Availability

The data analyzed in this study is subject to the following licenses/restrictions: dataset from epidemiological surveillance databases. Requests to access these datasets should be directed to rprieto@aspb.cat.
